# Supplementary figures and images for: Socio-economic factors and management regimes as drivers of tree cover change in Nepal
Source: PeerJ. 2018 May 29;6:e4855. doi: 10.7717/peerj.4855 (PMC5983000; doi:10.7717/peerj.4855)

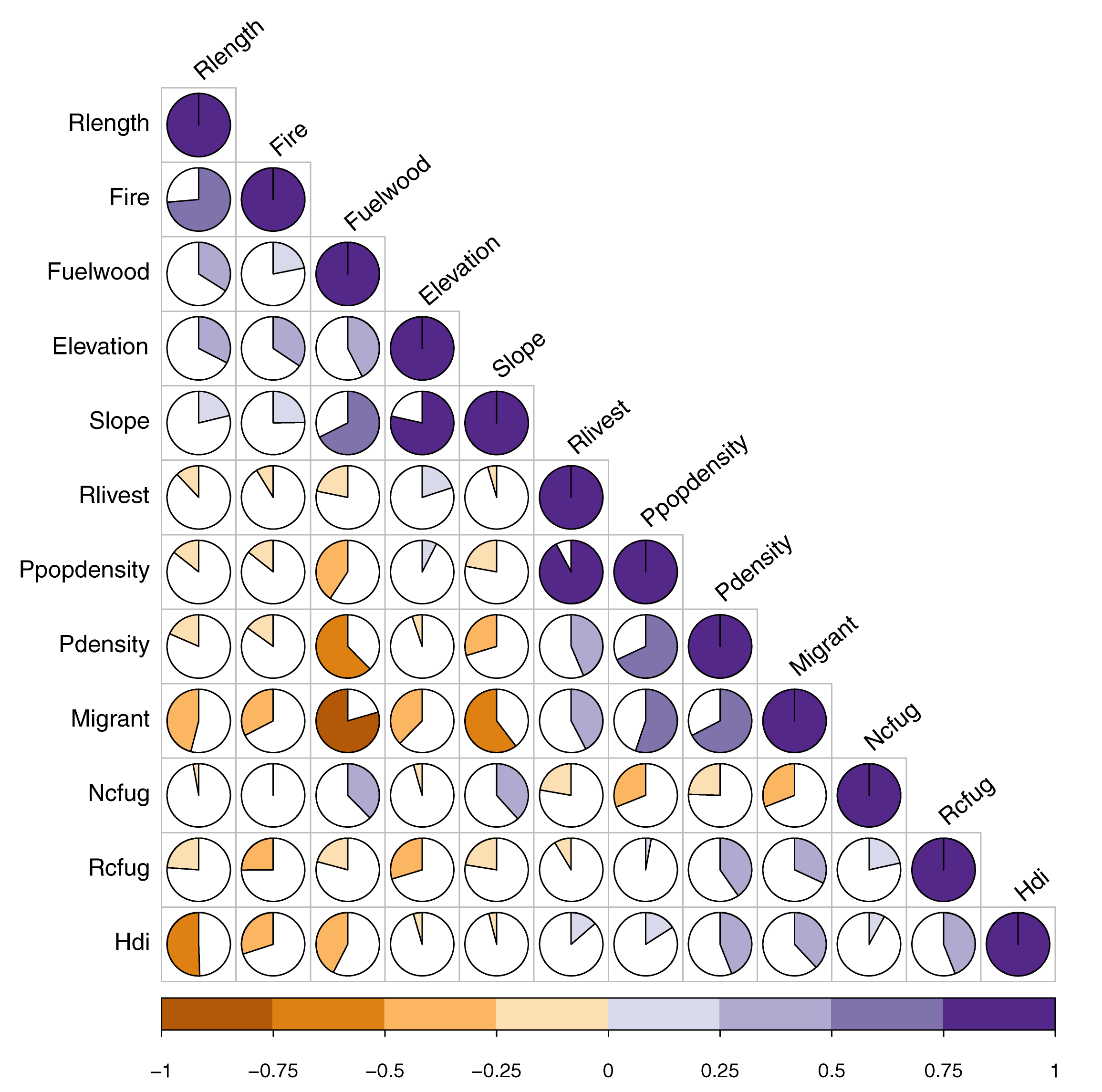

Supplement: Figure S1 [file peerj-06-4855-s001.png]

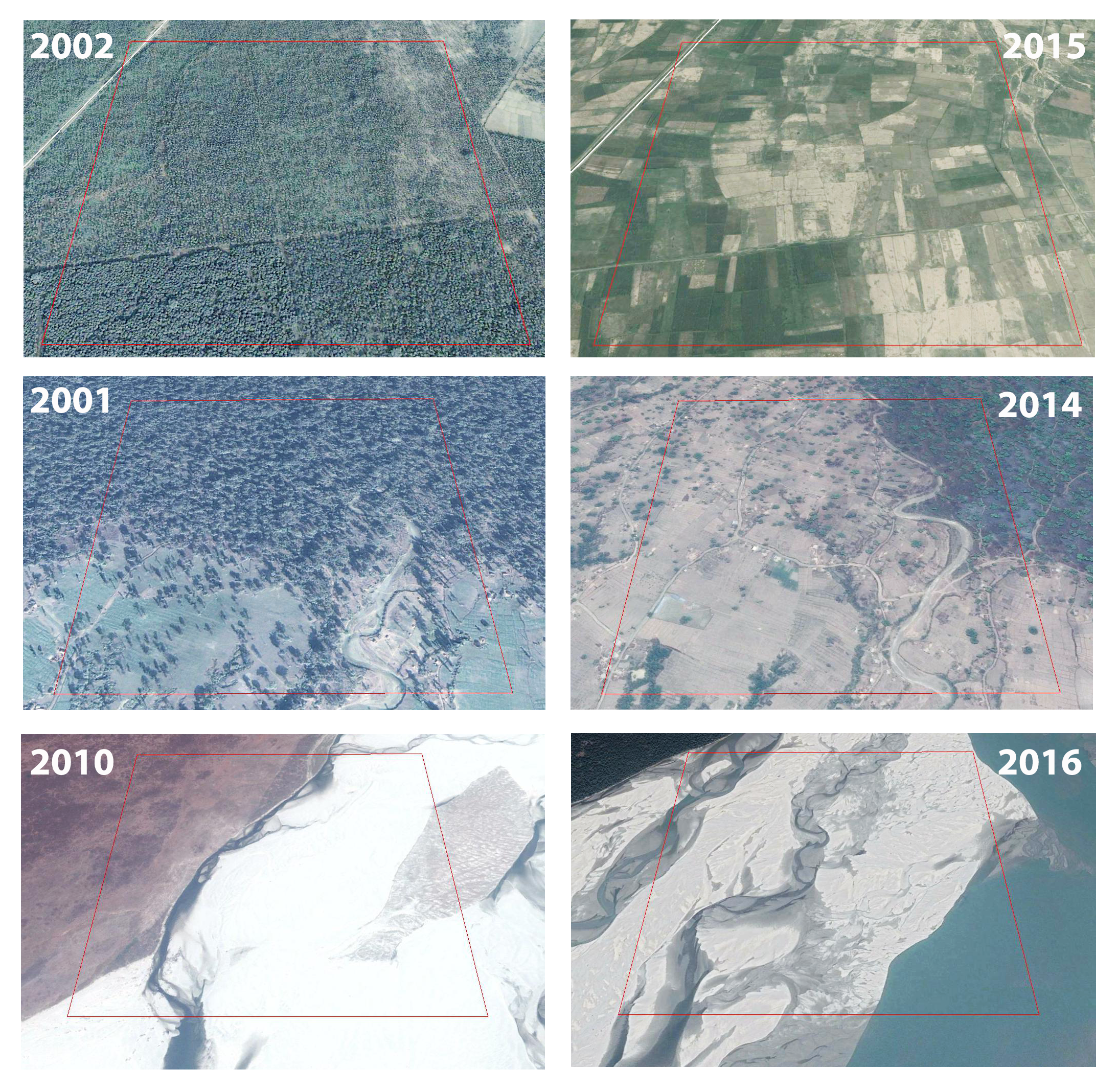

Supplement: Figure S2 [file peerj-06-4855-s002.png]
